# Supplementary material for: Inhibitory effect and mechanism of action (MOA) of hirsutine on the proliferation of T-cell leukemia Jurkat clone E6-1 cells
Source: PeerJ. 2021 Feb 2;9:e10692. doi: 10.7717/peerj.10692 (PMC7863788; doi:10.7717/peerj.10692)
Supplement: Supplemental Information 1 [file peerj-09-10692-s001.docx]

Raw data of CCK8 experiment (Figure 2)

Layout

Rawdata

| Raw data_Plate1 | | | | | |  |  |  |  |  |  |  |
| --- | --- | --- | --- | --- | --- | --- | --- | --- | --- | --- | --- | --- |
|  | 1 | 2 | 3 | 4 | 5 | 6 | 7 | 8 | 9 | 10 | 11 | 12 |
| A |  |  |  |  |  |  |  |  |  |  |  |  |
| B |  | 0.221 | 0.205 | 0.31 | 0.505 | 0.670 | 0.971 | 0.948 | 1.031 | 0.216 | 0.161 |  |
| C |  | 0.225 | 0.199 | 0.35 | 0.561 | 0.670 | 0.968 | 0.983 | 0.973 | 0.228 | 0.159 |  |
| D |  | 0.238 | 0.256 | 0.33 | 0.572 | 0.765 | 0.983 | 0.961 | 0.939 | 0.22 | 0.162 |  |
| E |  |  |  |  |  |  |  |  |  | 1.004 |  |  |
| F |  |  |  |  |  |  |  |  |  | 1.07 |  |  |
| G |  |  |  |  |  |  |  |  |  | 1.041 |  |  |
| H |  |  |  |  |  |  |  |  |  |  |  |  |

| Raw data_Plate2 | | | | | | | | | |  |  | |  | |  | |  | |  | |  | |
| --- | --- | --- | --- | --- | --- | --- | --- | --- | --- | --- | --- | --- | --- | --- | --- | --- | --- | --- | --- | --- | --- | --- |
|  | 1 | | 2 | | 3 | | 4 | | 5 | 6 | 7 | | 8 | | 9 | | 10 | | 11 | | 12 | |
| A |  | |  | |  | |  | |  |  |  | |  | |  | |  | |  | |  | |
| B |  | | 0.173 | | 0.153 | | 0.21 | | 0.306 | 0.759 | 0.808 | | 0.933 | | 0.971 | | 0.194 | | 0.126 | |  | |
| C |  | | 0.197 | | 0.164 | | 0.22 | | 0.313 | 0.746 | 0.753 | | 0.862 | | 0.976 | | 0.214 | | 0.124 | |  | |
| D |  | | 0.121 | | 0.114 | | 0.15 | | 0.231 | 0.697 | 0.767 | | 0.936 | | 1.086 | | 0.176 | | 0.124 | |  | |
| E |  | |  | |  | |  | |  |  |  | |  | |  | | 1.156 | |  | |  | |
| F |  | |  | |  | |  | |  |  |  | |  | |  | | 0.909 | |  | |  | |
| G |  | |  | |  | |  | |  |  |  | |  | |  | | 0.923 | |  | |  | |
| H |  | |  | |  | |  | |  |  |  | |  | |  | |  | |  | |  | |
| Raw data_Plate3 | | | | | | | | | |  |  |  | |  | |  | |  | |  | |  |
|  | | 1 | | 2 | | 3 | | 4 | 5 | 6 | 7 | 8 | | 9 | | 10 | | 11 | | 12 | |  |
| A | |  | |  | |  | |  |  |  |  |  | |  | |  | |  | |  | |  |
| B | |  | | 0.201 | | 0.205 | | 0.23 | 0.329 | 0.682 | 0.999 | 1.158 | | 1.103 | | 0.175 | | 0.151 | |  | |  |
| C | |  | | 0.188 | | 0.247 | | 0.23 | 0.316 | 0.867 | 1.122 | 1.21 | | 1.156 | | 0.233 | | 0.142 | |  | |  |
| D | |  | | 0.145 | | 0.148 | | 0.19 | 0.314 | 0.918 | 1.078 | 1.373 | | 1.334 | | 0.244 | | 0.142 | |  | |  |
| E | |  | |  | |  | |  |  |  |  |  | |  | | 1.394 | |  | |  | |  |
| F | |  | |  | |  | |  |  |  |  |  | |  | | 1.289 | |  | |  | |  |
| G | |  | |  | |  | |  |  |  |  |  | |  | | 1.119 | |  | |  | |  |
| H | |  | |  | |  | |  |  |  |  |  | |  | |  | |  | |  | |  |

| Raw Data_plate1 Jurkat E6-1 | | | | | | | |  |  | |  | |  | |  | |  | |  | |  | |  | |  |
| --- | --- | --- | --- | --- | --- | --- | --- | --- | --- | --- | --- | --- | --- | --- | --- | --- | --- | --- | --- | --- | --- | --- | --- | --- | --- |
|  | | 1 | 2 | | 3 | | | 4 | 5 | | 6 | | 7 | | 8 | | 9 | | 10 | | 11 | | 12 | |  |
| A | |  |  | |  | | |  |  | |  | |  | |  | |  | |  | |  | |  | |  |
| B | |  | 0.221 | | 0.205 | | | 0.313 | 0.505 | | 0.67 | | 0.971 | | 0.948 | | 1.031 | | 0.216 | | 0.161 | |  | |  |
| C | |  | 0.225 | | 0.199 | | | 0.346 | 0.561 | | 0.67 | | 0.968 | | 0.983 | | 0.973 | | 0.228 | | 0.159 | |  | |  |
| D | |  | 0.238 | | 0.256 | | | 0.331 | 0.572 | | 0.765 | | 0.983 | | 0.961 | | 0.939 | | 0.22 | | 0.162 | |  | |  |
| E | |  |  | |  | | |  |  | |  | |  | |  | |  | | 1.004 | |  | |  | |  |
| F | |  |  | |  | | |  |  | |  | |  | |  | |  | | 1.07 | |  | |  | |  |
| G | |  |  | |  | | |  |  | |  | |  | |  | |  | | 1.041 | |  | |  | |  |
| H | |  |  | |  | | |  |  | |  | |  | |  | |  | |  | |  | |  | |  |
|  | |  |  | |  | | |  |  | |  | |  | |  | |  | |  | | Blank | |  | |  |
|  | |  |  | |  | | |  |  | |  | |  | |  | |  | |  | | 0.16 | |  | |  |
|  | 1 | | | 2 | | 3 | 4 | | | 5 | | 6 | | 7 | | 8 | | 9 | | 10 | | 11 | | 12 | |
| A |  | | |  | |  |  | | |  | |  | |  | |  | |  | |  | |  | |  | |
| B |  | | | 0.060 | | 0.044 | 0.152 | | | 0.344 | | 0.509 | | 0.810 | | 0.787 | | 0.870 | | 0.055 | | 0.000 | |  | |
| C |  | | | 0.064 | | 0.038 | 0.185 | | | 0.400 | | 0.509 | | 0.807 | | 0.822 | | 0.812 | | 0.067 | | -0.002 | |  | |
| D |  | | | 0.077 | | 0.095 | 0.170 | | | 0.411 | | 0.604 | | 0.822 | | 0.800 | | 0.778 | | 0.059 | | 0.001 | |  | |
| E |  | | |  | |  |  | | |  | |  | |  | |  | |  | | 0.843 | |  | |  | |
| F |  | | |  | |  |  | | |  | |  | |  | |  | |  | | 0.909 | |  | |  | |
| G |  | | |  | |  |  | | |  | |  | |  | |  | |  | | 0.880 | |  | |  | |
| H |  | | |  | |  |  | | |  | |  | |  | |  | |  | |  | |  | |  | |
|  |  | | |  | |  |  | | |  | |  | |  | |  | |  | |  | | Neg.Ctrl | |  | |
|  |  | | |  | |  |  | | |  | |  | |  | |  | |  | |  | | 0.878 | |  | |

| Cell growth% | | |  |  |  |  |  |  |  |  |  |  |
| --- | --- | --- | --- | --- | --- | --- | --- | --- | --- | --- | --- | --- |
|  | 1 | 2 | 3 | 4 | 5 | 6 | 7 | 8 | 9 | 10 | 11 | 12 |
| A |  |  |  |  |  |  |  |  |  |  |  |  |
| B |  | 6.87 | 5.05 | 17.36 | 39.23 | 58.03 | 92.33 | 89.71 | 99.16 | 6.30 | 0.04 |  |
| C |  | 7.33 | 4.37 | 21.12 | 45.61 | 58.03 | 91.99 | 93.70 | 92.56 | 7.67 | -0.19 |  |
| D |  | 8.81 | 10.86 | 19.41 | 46.87 | 68.86 | 93.70 | 91.19 | 88.68 | 6.76 | 0.15 |  |
| E |  |  |  |  |  |  |  |  |  | 96.09 |  |  |
| F |  |  |  |  |  |  |  |  |  | 103.61 |  |  |
| G |  |  |  |  |  |  |  |  |  | 100.30 |  |  |
| H |  |  |  |  |  |  |  |  |  |  |  |  |

| **Compound ID** | **IC_50_** |
| --- | --- |
| hirsutine | 29.40μM |

| Raw Data_plate2 Jurkat E6-1 | | | | | | | |  | |  |  |  |  |  |  |  |  |
| --- | --- | --- | --- | --- | --- | --- | --- | --- | --- | --- | --- | --- | --- | --- | --- | --- | --- |
|  | 1 | | 2 | | 3 | | | 4 | | 5 | 6 | 7 | 8 | 9 | 10 | 11 | 12 |
| A |  | |  | |  | | |  | |  |  |  |  |  |  |  |  |
| B |  | | 0.173 | | 0.153 | | | 0.211 | | 0.306 | 0.759 | 0.808 | 0.933 | 0.971 | 0.194 | 0.126 |  |
| C |  | | 0.197 | | 0.164 | | | 0.219 | | 0.313 | 0.746 | 0.753 | 0.862 | 0.976 | 0.214 | 0.124 |  |
| D |  | | 0.121 | | 0.114 | | | 0.15 | | 0.231 | 0.697 | 0.767 | 0.936 | 1.086 | 0.176 | 0.124 |  |
| E |  | |  | |  | | |  | |  |  |  |  |  | 1.156 |  |  |
| F |  | |  | |  | | |  | |  |  |  |  |  | 0.909 |  |  |
| G |  | |  | |  | | |  | |  |  |  |  |  | 0.923 |  |  |
| H |  | |  | |  | | |  | |  |  |  |  |  |  |  |  |
|  |  | |  | |  | | |  | |  |  |  |  |  |  | \| Blank \| \| --- \| \| 0.12 \| |  |
|  | | 1 | | 2 | | 3 | 4 | | 5 | | 6 | 7 | 8 | 9 | 10 | 11 | 12 |
| A | |  | |  | |  |  | |  | |  |  |  |  |  |  |  |
| B | |  | | 0.048 | | 0.028 | 0.086 | | 0.181 | | 0.634 | 0.683 | 0.808 | 0.846 | 0.069 | 0.001 |  |
| C | |  | | 0.072 | | 0.039 | 0.094 | | 0.188 | | 0.621 | 0.628 | 0.737 | 0.851 | 0.089 | -0.001 |  |
| D | |  | | -0.004 | | -0.011 | 0.025 | | 0.106 | | 0.572 | 0.642 | 0.811 | 0.961 | 0.051 | -0.001 |  |
| E | |  | |  | |  |  | |  | |  |  |  |  | 1.031 |  |  |
| F | |  | |  | |  |  | |  | |  |  |  |  | 0.784 |  |  |
| G | |  | |  | |  |  | |  | |  |  |  |  | 0.798 |  |  |
| H | |  | |  | |  |  | |  | |  |  |  |  |  |  |  |
|  | |  | |  | |  |  | |  | |  |  |  |  |  | Neg.Ctrl |  |
|  | |  | |  | |  |  | |  | |  |  |  |  |  | 0.871 |  |

| Cell growth% | | |  |  |  |  |  |  |  |  |  |  |
| --- | --- | --- | --- | --- | --- | --- | --- | --- | --- | --- | --- | --- |
|  | 1 | 2 | 3 | 4 | 5 | 6 | 7 | 8 | 9 | 10 | 11 | 12 |
| A |  |  |  |  |  |  |  |  |  |  |  |  |
| B |  | 5.55 | 3.25 | 9.91 | 20.81 | 72.80 | 78.42 | 92.77 | 97.13 | 7.96 | 0.15 |  |
| C |  | 8.30 | 4.51 | 10.83 | 21.61 | 71.31 | 72.11 | 84.62 | 97.70 | 10.25 | -0.08 |  |
| D |  | -0.42 | -1.22 | 2.91 | 12.20 | 65.68 | 73.72 | 93.11 | 110.33 | 5.89 | -0.08 |  |
| E |  |  |  |  |  |  |  |  |  | 118.36 |  |  |
| F |  |  |  |  |  |  |  |  |  | 90.02 |  |  |
| G |  |  |  |  |  |  |  |  |  | 91.62 |  |  |
| H |  |  |  |  |  |  |  |  |  |  |  |  |

| **Compound ID** | **IC_50_** |
| --- | --- |
| hirsutine | 24.18μM |

| Raw Data_plate3 Jurkat E6-1 | | |  |  |  |  |  |  |  |  |  |  |
| --- | --- | --- | --- | --- | --- | --- | --- | --- | --- | --- | --- | --- |
|  | 1 | 2 | 3 | 4 | 5 | 6 | 7 | 8 | 9 | 10 | 11 | 12 |
| A |  |  |  |  |  |  |  |  |  |  |  |  |
| B |  | 0.201 | 0.205 | 0.229 | 0.329 | 0.682 | 0.999 | 1.158 | 1.103 | 0.175 | 0.151 |  |
| C |  | 0.188 | 0.247 | 0.226 | 0.316 | 0.867 | 1.122 | 1.21 | 1.156 | 0.233 | 0.142 |  |
| D |  | 0.145 | 0.148 | 0.188 | 0.314 | 0.918 | 1.078 | 1.373 | 1.334 | 0.244 | 0.142 |  |
| E |  |  |  |  |  |  |  |  |  | 1.394 |  |  |
| F |  |  |  |  |  |  |  |  |  | 1.289 |  |  |
| G |  |  |  |  |  |  |  |  |  | 1.119 |  |  |
| H |  |  |  |  |  |  |  |  |  |  |  |  |
|  |  |  |  |  |  |  |  |  |  |  | Blank |  |
|  |  |  |  |  |  |  |  |  |  |  | 0.15 |  |

|  | 1 | 2 | | | 3 | | 4 | | 5 | | 6 | | 7 | | 8 | | 9 | | 10 | | 11 | | 12 |
| --- | --- | --- | --- | --- | --- | --- | --- | --- | --- | --- | --- | --- | --- | --- | --- | --- | --- | --- | --- | --- | --- | --- | --- |
| A |  |  | | |  | |  | |  | |  | |  | |  | |  | |  | |  | |  |
| B |  | 0.056 | | | 0.060 | | 0.084 | | 0.184 | | 0.537 | | 0.854 | | 1.013 | | 0.958 | | 0.030 | | 0.006 | |  |
| C |  | 0.043 | | | 0.102 | | 0.081 | | 0.171 | | 0.722 | | 0.977 | | 1.065 | | 1.011 | | 0.088 | | -0.003 | |  |
| D |  | 0.000 | | | 0.003 | | 0.043 | | 0.169 | | 0.773 | | 0.933 | | 1.228 | | 1.189 | | 0.099 | | -0.003 | |  |
| E |  |  | | |  | |  | |  | |  | |  | |  | |  | | 1.249 | |  | |  |
| F |  |  | | |  | |  | |  | |  | |  | |  | |  | | 1.144 | |  | |  |
| G |  |  | | |  | |  | |  | |  | |  | |  | |  | | 0.974 | |  | |  |
| H |  |  | | |  | |  | |  | |  | |  | |  | |  | |  | |  | |  |
|  |  |  | | |  | |  | |  | |  | |  | |  | |  | |  | | Neg.Ctrl | |  |
|  |  |  | | |  | |  | |  | |  | |  | |  | |  | |  | | 1.122 | |  |
| Cell growth% | | | |  | |  | |  | |  | |  | |  | |  | |  | |  | |  |  |
|  | | | 1 | 2 | | 3 | | 4 | | 5 | | 6 | | 7 | | 8 | | 9 | | 10 | | 11 | 12 |
| A | | |  |  | |  | |  | |  | |  | |  | |  | |  | |  | |  |  |
| B | | |  | 4.99 | | 5.35 | | 7.48 | | 16.39 | | 47.85 | | 76.09 | | 90.26 | | 85.36 | | 2.67 | | 0.53 |  |
| C | | |  | 3.83 | | 9.09 | | 7.22 | | 15.24 | | 64.33 | | 87.05 | | 94.89 | | 90.08 | | 7.84 | | -0.27 |  |
| D | | |  | 0.00 | | 0.27 | | 3.83 | | 15.06 | | 68.87 | | 83.13 | | 109.41 | | 105.94 | | 8.82 | | -0.27 |  |
| E | | |  |  | |  | |  | |  | |  | |  | |  | |  | | 111.29 | |  |  |
| F | | |  |  | |  | |  | |  | |  | |  | |  | |  | | 101.93 | |  |  |
| G | | |  |  | |  | |  | |  | |  | |  | |  | |  | | 86.78 | |  |  |
| H | | |  |  | |  | |  | |  | |  | |  | |  | |  | |  | |  |  |

| **Compound ID** | **IC_50_** |
| --- | --- |
| hirsutine | 21.33μM |

| **Compound ID** | **24h-IC_50_** | **48h-IC_50_** | **72h-IC_50_** |
| --- | --- | --- | --- |
| hirsutine | 29.40μM | 24.18μM | 21.33μM |

Normal cells

Layout


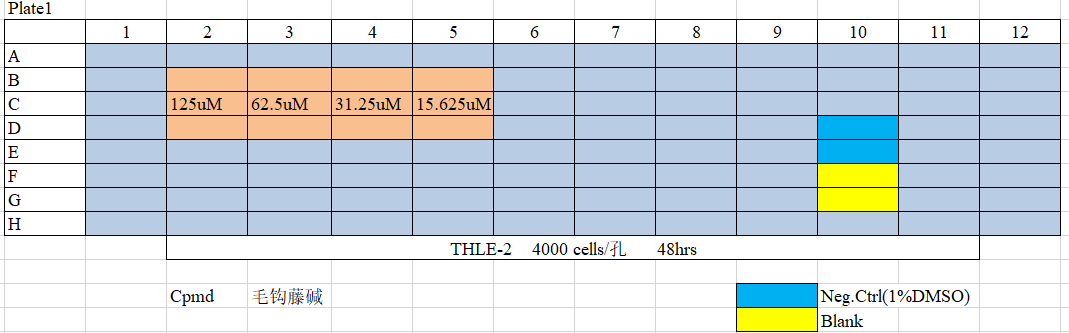

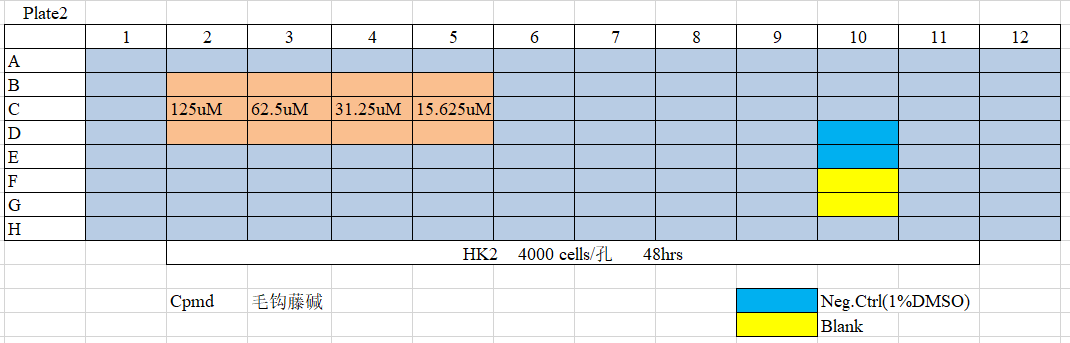


| Raw data_Plate1 | | | | | |  |  |  |  |  |  |  |
| --- | --- | --- | --- | --- | --- | --- | --- | --- | --- | --- | --- | --- |
|  | 1 | 2 | 3 | 4 | 5 | 6 | 7 | 8 | 9 | 10 | 11 | 12 |
| A |  |  |  |  |  |  |  |  |  |  |  |  |
| B |  | 0.802 | 0.943 | 1.071 | 1.166 |  |  |  |  |  |  |  |
| C |  | 0.889 | 1.069 | 1.195 | 1.290 |  |  |  |  |  |  |  |
| D |  | 0.865 | 1.046 | 1.209 | 1.297 |  |  |  |  | 1.322 |  |  |
| E |  |  |  |  |  |  |  |  |  | 1.269 |  |  |
| F |  |  |  |  |  |  |  |  |  | 0.106 |  |  |
| G |  |  |  |  |  |  |  |  |  | 0.103 |  |  |
| H |  |  |  |  |  |  |  |  |  |  |  |  |
|  |  | THLE-2 4000 cells/孔 48hrs | | | | | | | | | |  |

| Raw data_Plate2 | | | | | |  |  |  |  |  |  |  |
| --- | --- | --- | --- | --- | --- | --- | --- | --- | --- | --- | --- | --- |
|  | 1 | 2 | 3 | 4 | 5 | 6 | 7 | 8 | 9 | 10 | 11 | 12 |
| A |  |  |  |  |  |  |  |  |  |  |  |  |
| B |  | 1.282 | 1.332 | 1.282 | 1.266 |  |  |  |  |  |  |  |
| C |  | 1.349 | 1.387 | 1.375 | 1.359 |  |  |  |  |  |  |  |
| D |  | 1.278 | 1.353 | 1.357 | 1.342 |  |  |  |  | 1.354 |  |  |
| E |  |  |  |  |  |  |  |  |  | 1.356 |  |  |
| F |  |  |  |  |  |  |  |  |  | 0.105 |  |  |
| G |  |  |  |  |  |  |  |  |  | 0.102 |  |  |
| H |  |  |  |  |  |  |  |  |  |  |  |  |
|  |  | HK2 4000 cells/孔 48hrs | | | | | | | | | |  |

| Cell toxic: %Control | | |  |  |  |  |  |  |  |  |  |  |
| --- | --- | --- | --- | --- | --- | --- | --- | --- | --- | --- | --- | --- |
|  | 1 | 2 | 3 | 4 | 5 | 6 | 7 | 8 | 9 | 10 | 11 | 12 |
| A |  |  |  |  |  |  |  |  |  |  |  |  |
| B |  | 58.564 | 70.403 | 81.150 | 89.127 |  |  |  |  |  |  |  |
| C |  | 65.869 | 80.982 | 91.562 | 99.538 |  |  |  |  |  |  |  |
| D |  | 63.854 | 79.051 | 92.737 | 100.126 |  |  |  |  | 102.225 |  |  |
| E |  |  |  |  |  |  |  |  |  | 97.775 |  |  |
| F |  |  |  |  |  |  |  |  |  | 0.126 |  |  |
| G |  |  |  |  |  |  |  |  |  | -0.126 |  |  |
| H |  |  |  |  |  |  |  |  |  |  |  |  |

| Cell toxic: %Control | |  |  |  |  |  |  |  |  |  |  |  |
| --- | --- | --- | --- | --- | --- | --- | --- | --- | --- | --- | --- | --- |
|  | 1 | 2 | 3 | 4 | 5 | 6 | 7 | 8 | 9 | 10 | 11 | 12 |
| A |  |  |  |  |  |  |  |  |  |  |  |  |
| B |  | 98.866 | 103.065 | 98.866 | 97.523 |  |  |  |  |  |  |  |
| C |  | 104.492 | 107.683 | 106.675 | 105.332 |  |  |  |  |  |  |  |
| D |  | 98.531 | 104.828 | 105.164 | 103.904 |  |  |  |  | 104.912 |  |  |
| E |  |  |  |  |  |  |  |  |  | 105.080 |  |  |
| F |  |  |  |  |  |  |  |  |  | 0.125 |  |  |
| G |  |  |  |  |  |  |  |  |  | -0.125 |  |  |
| H |  |  |  |  |  |  |  |  |  |  |  |  |
