# Supplementary material for: Inhibitory effect and mechanism of action (MOA) of hirsutine on the proliferation of T-cell leukemia Jurkat clone E6-1 cells
Source: PeerJ. 2021 Feb 2;9:e10692. doi: 10.7717/peerj.10692 (PMC7863788; doi:10.7717/peerj.10692)
Supplement: Supplemental Information 2 [file peerj-09-10692-s002.docx]

**Raw data of apoptosis experiment results**

1、DMSO group


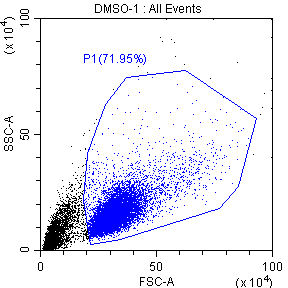

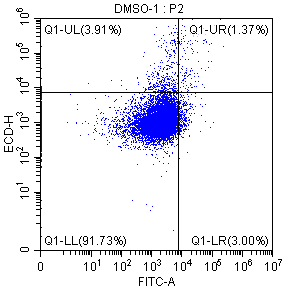

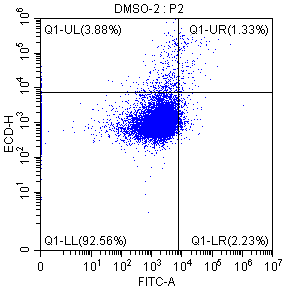

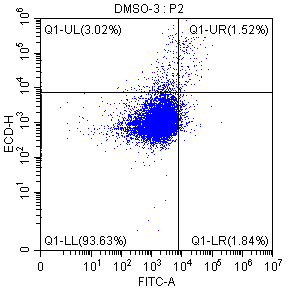


2、10 μM group


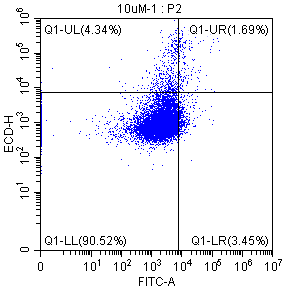

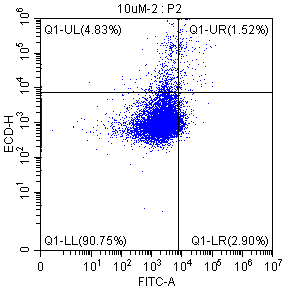

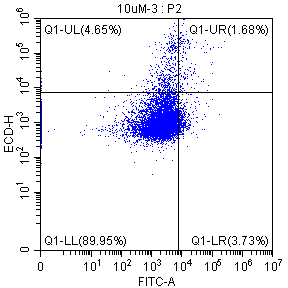


3、25 μM group


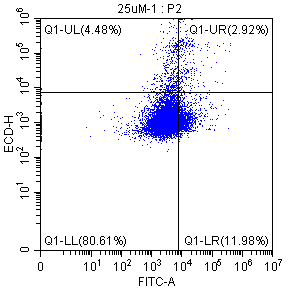

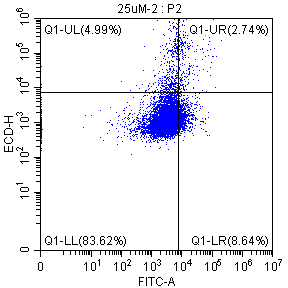

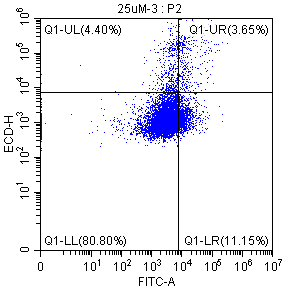


4、50 μM group


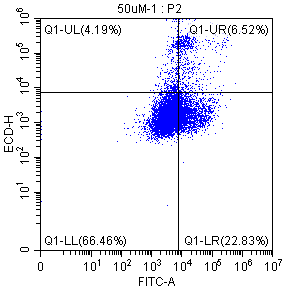

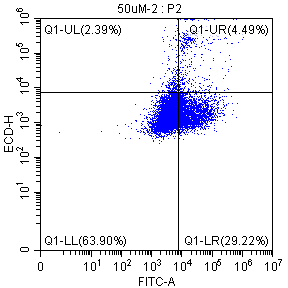

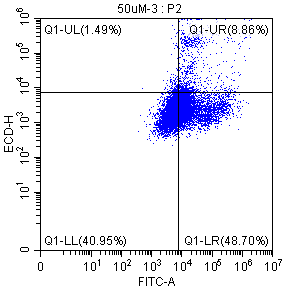


| **Group** | **Early apoptosis rate** | **Late apoptosis rate** | **Apoptosis rate** |
| --- | --- | --- | --- |
| DMSO | 3 | 1.37 | 4.37 |
|  | 2.23 | 1.33 | 3.56 |
|  | 1.84 | 1.52 | 3.36 |
| 10 μM | 3.45 | 1.69 | 5.14 |
|  | 2.9 | 1.52 | 4.42 |
|  | 3.73 | 1.68 | 5.41 |
| 25 μM | 11.98 | 2.92 | 14.9 |
|  | 8.64 | 2.74 | 11.38 |
|  | 11.15 | 3.65 | 14.8 |
| 50 μM | 22.83 | 6.52 | 29.35 |
|  | 29.22 | 4.49 | 33.71 |
|  | 48.7 | 8.86 | 57.56 |
