# Supplementary material for: Inhibitory effect and mechanism of action (MOA) of hirsutine on the proliferation of T-cell leukemia Jurkat clone E6-1 cells
Source: PeerJ. 2021 Feb 2;9:e10692. doi: 10.7717/peerj.10692 (PMC7863788; doi:10.7717/peerj.10692)

Raw data of cell cycle experiment results

Cell cycle distribution percentage

| Group | G1 | G2 | S |
| --- | --- | --- | --- |
| DMSO-1 | 22.74 | 1.17 | 76.09 |
| DMSO-2 | 15.67 | 2.51 | 81.82 |
| DMSO-3 | 23.22 | 0.11 | 76.78 |
| 10 μM-1 | 32.77 | 1.11 | 66.12 |
| 10 μM -2 | 36.07 | 0 | 63.93 |
| 10 μM -3 | 35.7 | 0 | 64.3 |
| 25 μM -1 | 42.36 | 3.98 | 53.67 |
| 25 μM -2 | 43.63 | 1.21 | 55.15 |
| 25 μM -3 | 42.5 | 2.26 | 55.24 |
| 50 μM -1 | 45.69 | 0.51 | 54.31 |
| 50 μM -2 | 48.05 | 1.69 | 50.25 |
| 50 μM -3 | 53.61 | 0.32 | 46.39 |

DMSO-1：


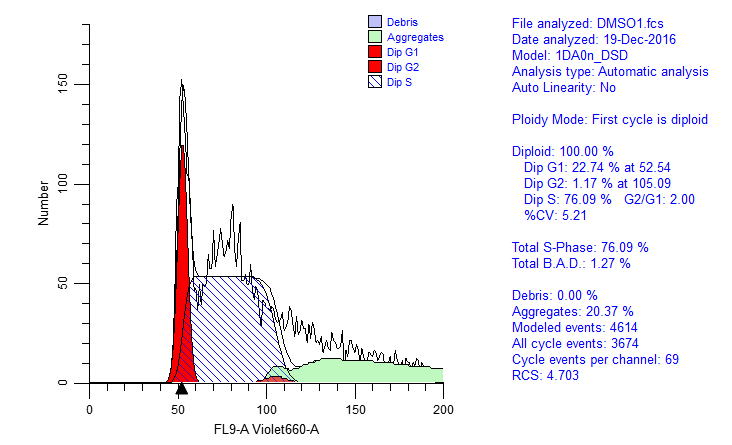


DMSO-2：


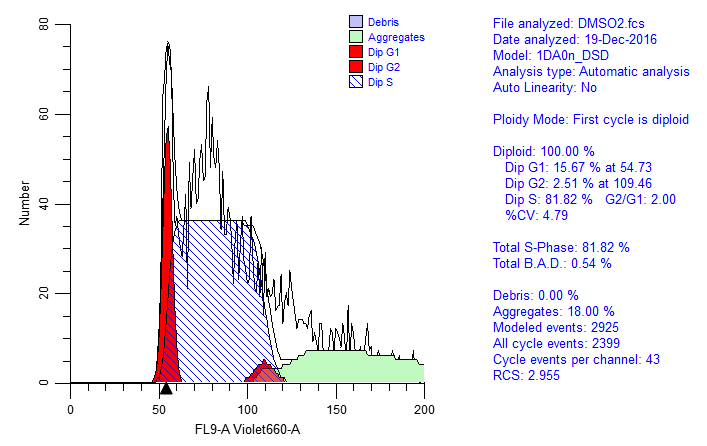


DMSO-3：


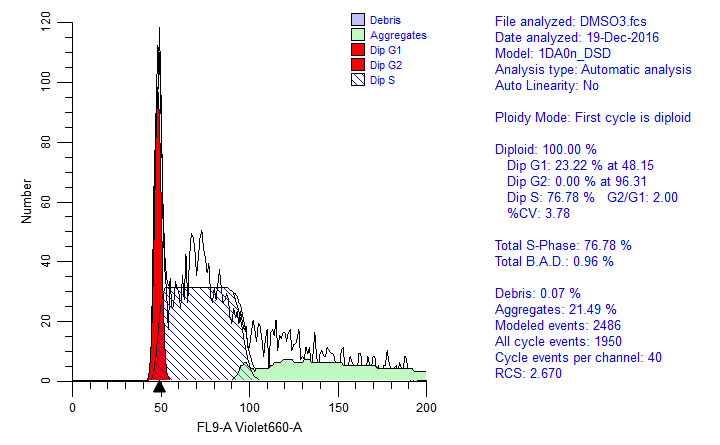


10 μM-1：


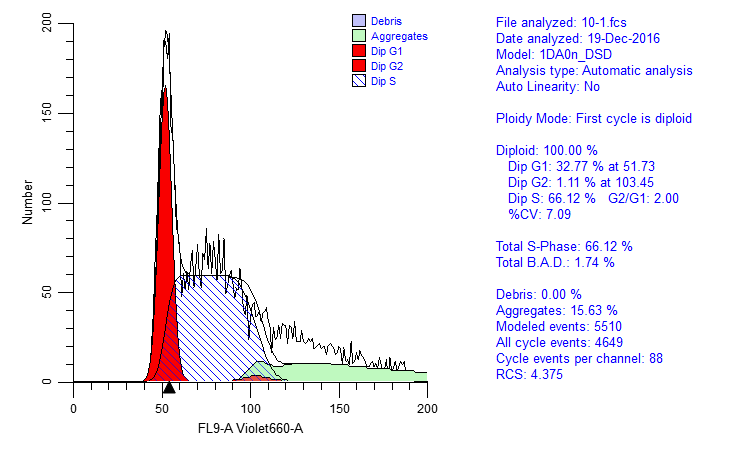


10 μM -2：


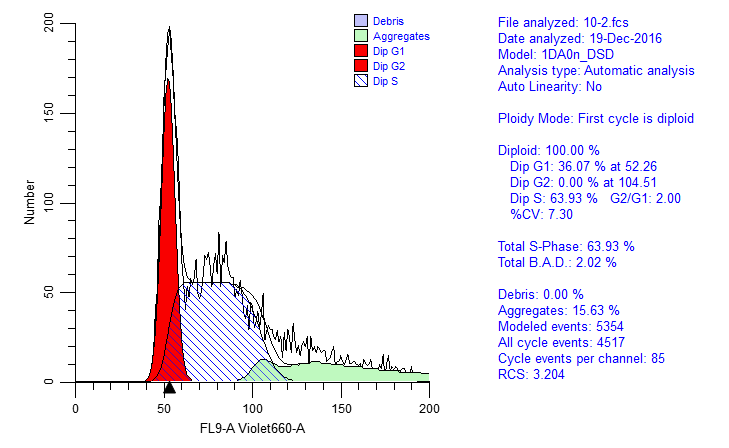


10 μM -3：


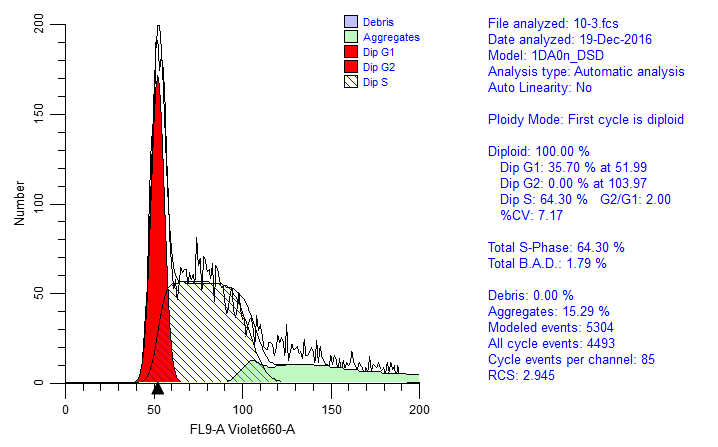


25 μM -1：


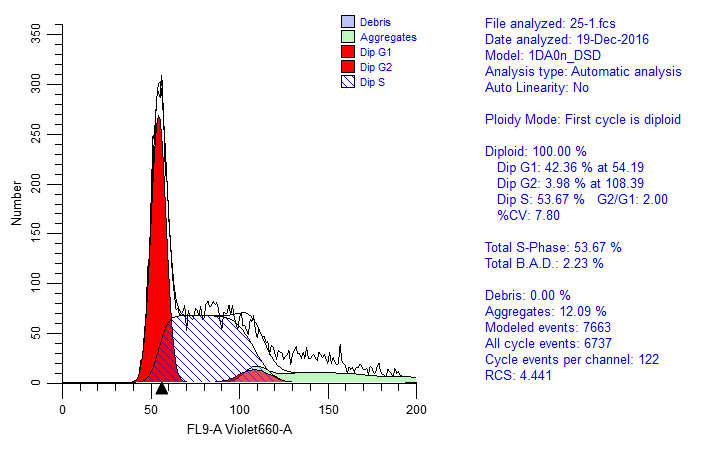


25 μM -2：


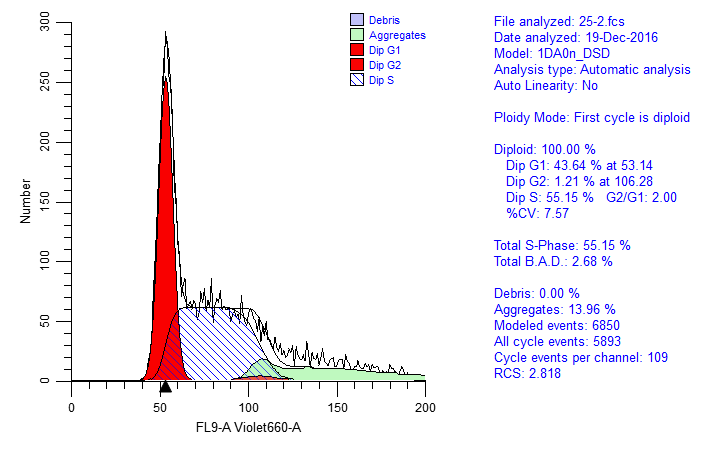


25 μM -3：


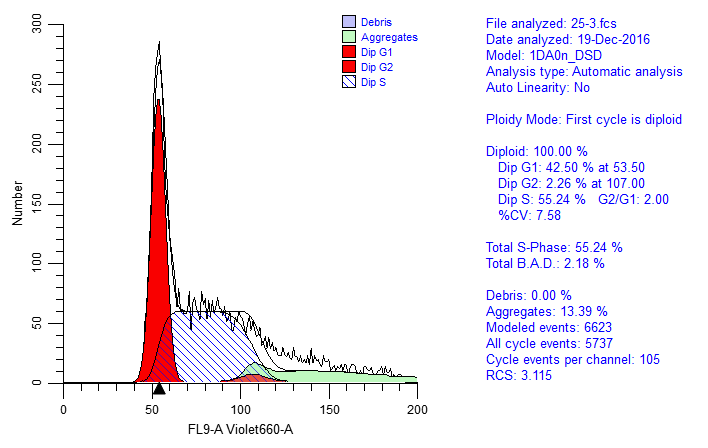


50 μM -1：


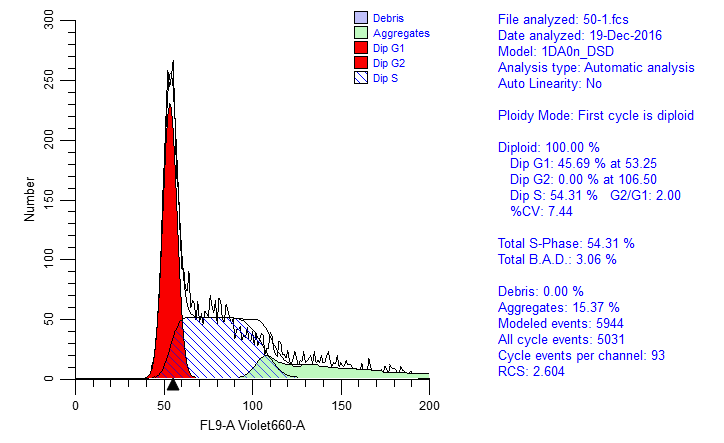


50 μM -2：


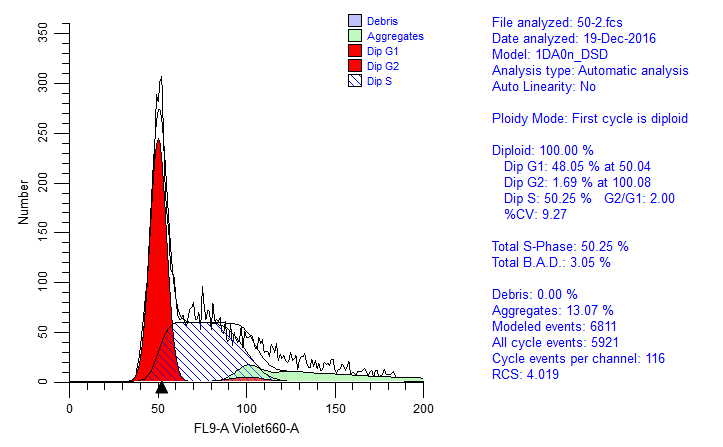


50 μM -3：


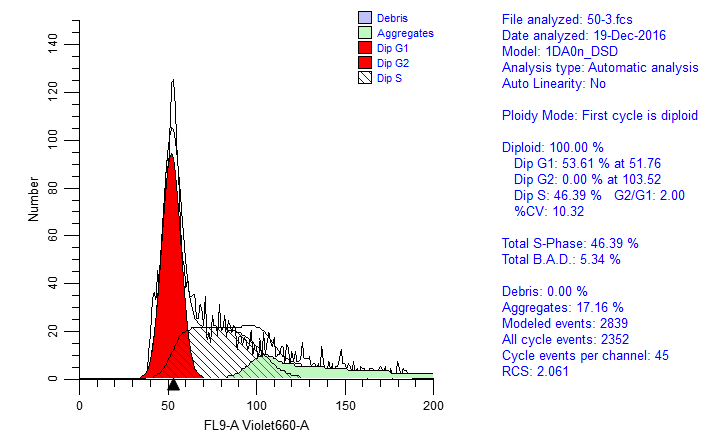

Supplement: Supplemental Information 3 [file peerj-09-10692-s003.docx]
