# Supplementary figures and images for: Inhibitory effect and mechanism of action (MOA) of hirsutine on the proliferation of T-cell leukemia Jurkat clone E6-1 cells
Source: PeerJ. 2021 Feb 2;9:e10692. doi: 10.7717/peerj.10692 (PMC7863788; doi:10.7717/peerj.10692)

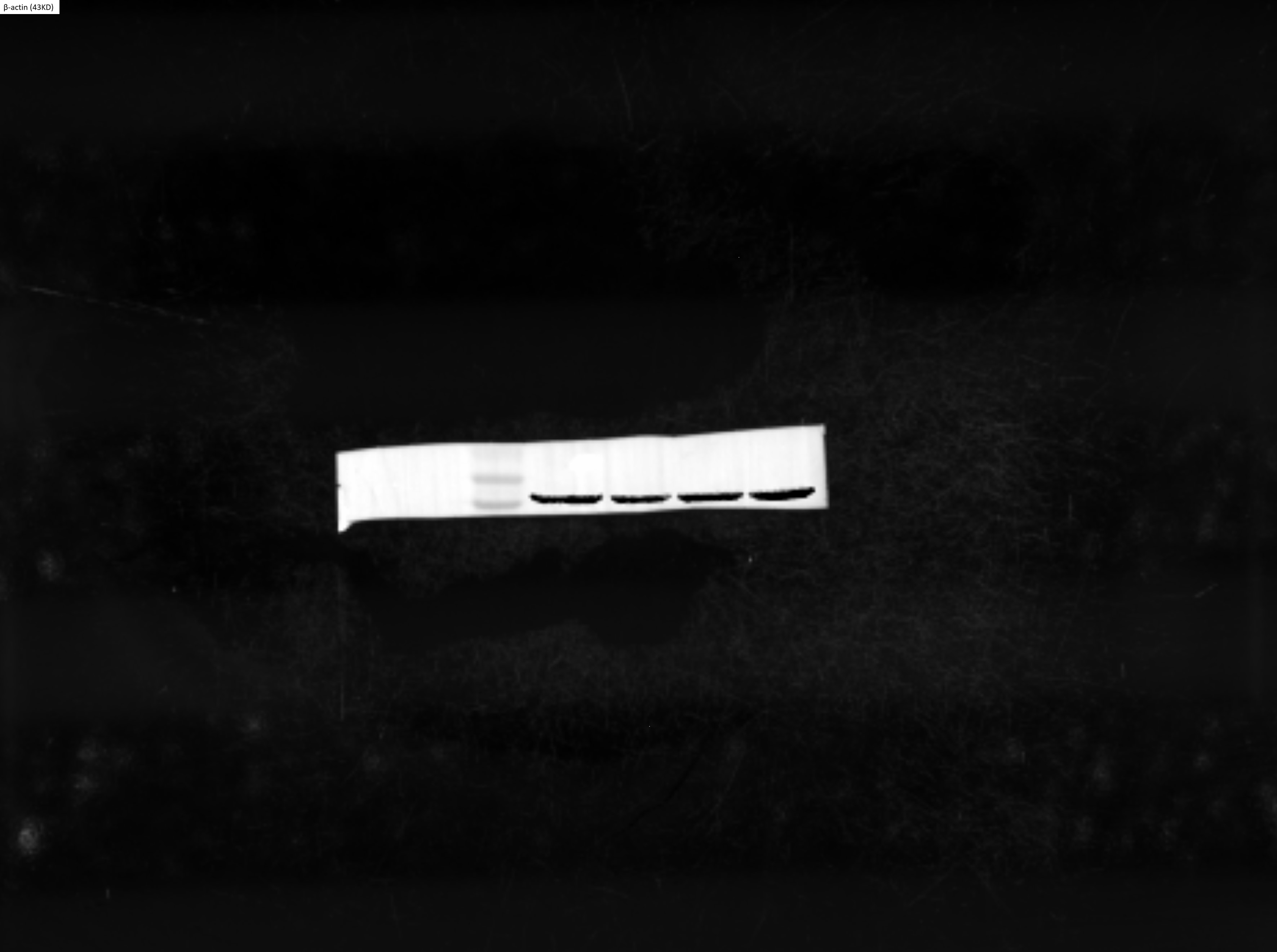

Supplement: Supplemental Information 4 [file peerj-09-10692-s004.zip › actin-1.jpg]

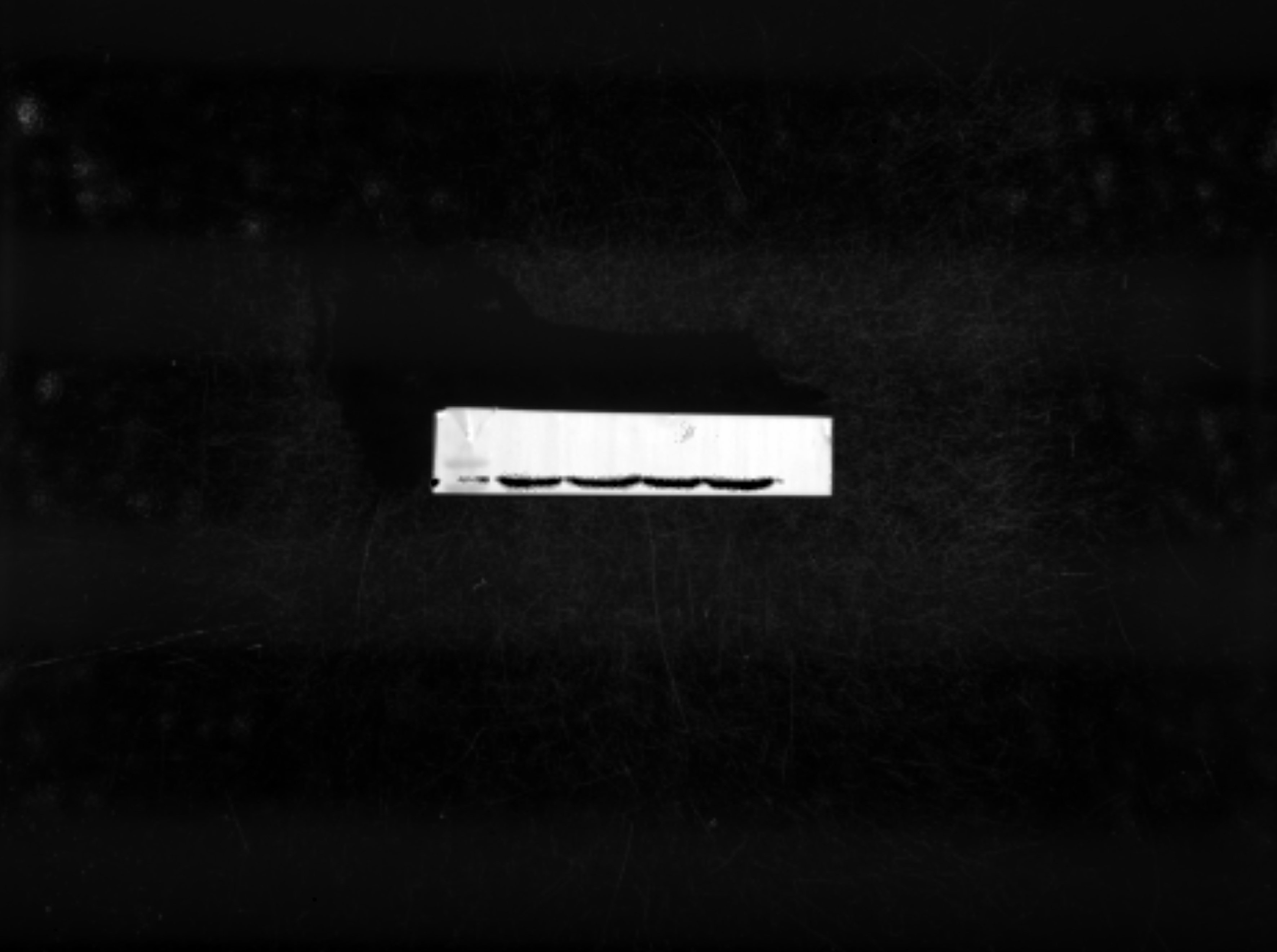

Supplement: Supplemental Information 4 [file peerj-09-10692-s004.zip › actin-2.jpg]

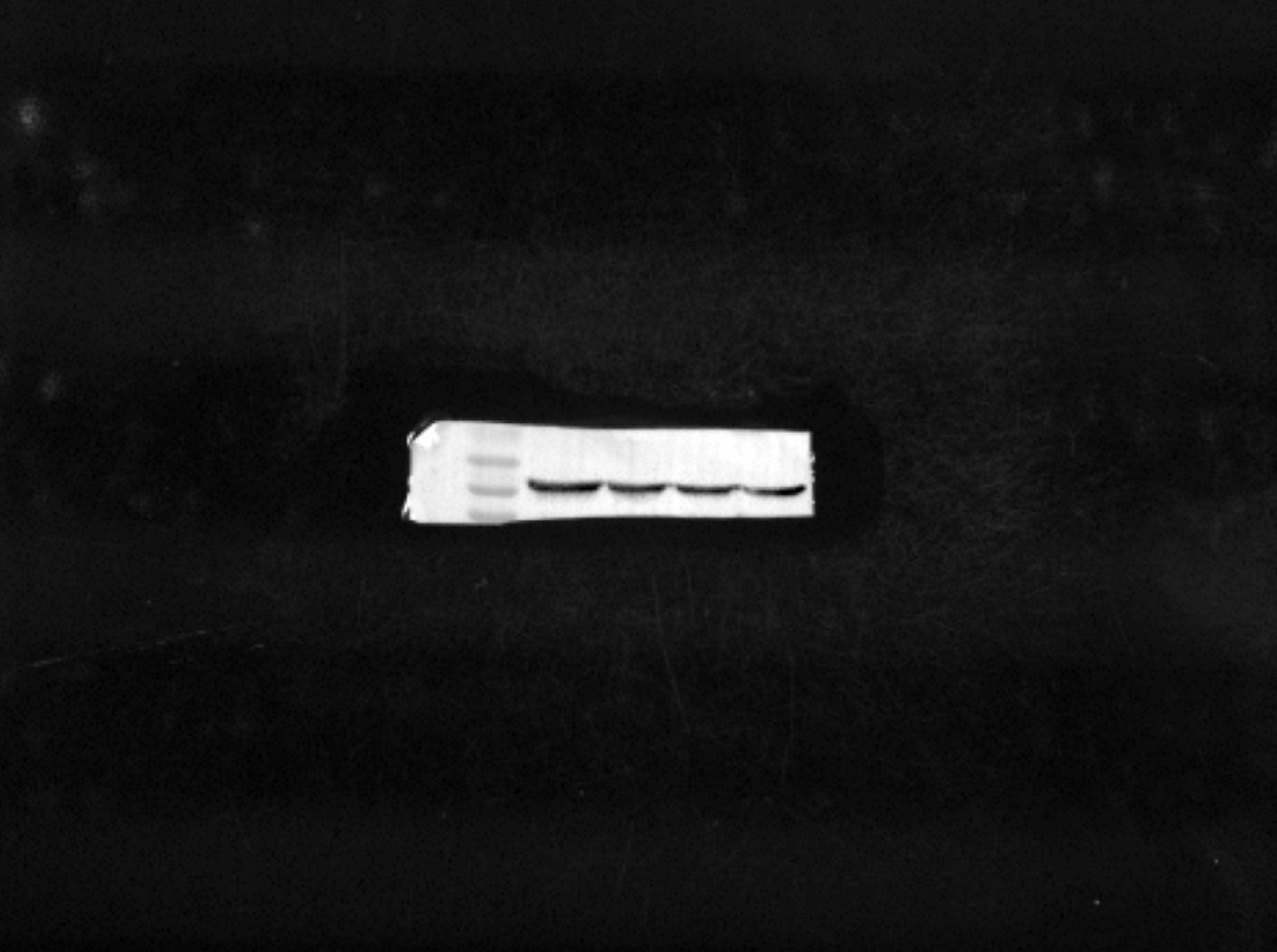

Supplement: Supplemental Information 4 [file peerj-09-10692-s004.zip › actin-3.jpg]

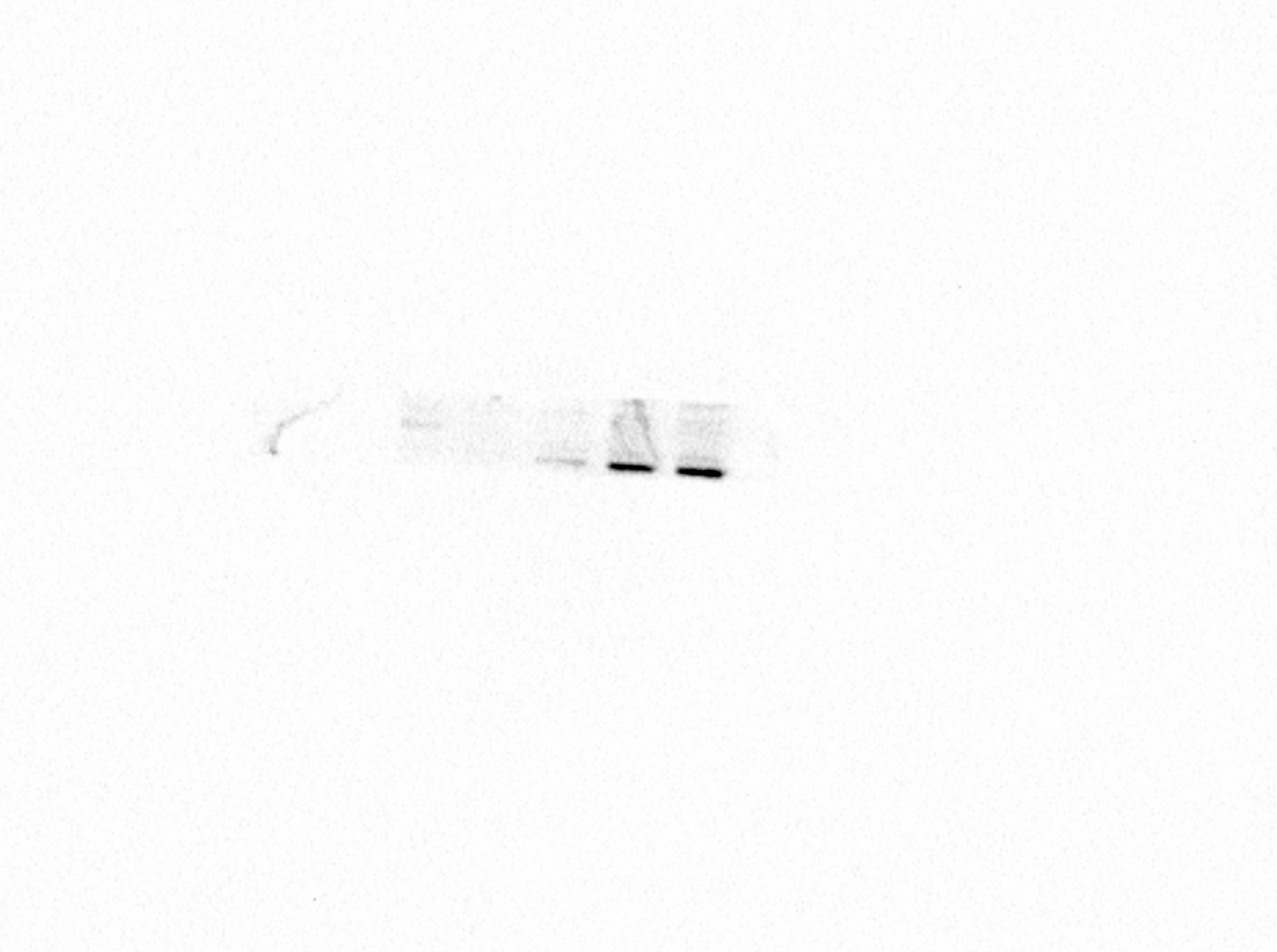

Supplement: Supplemental Information 4 [file peerj-09-10692-s004.zip › BCL-2-1.jpg]

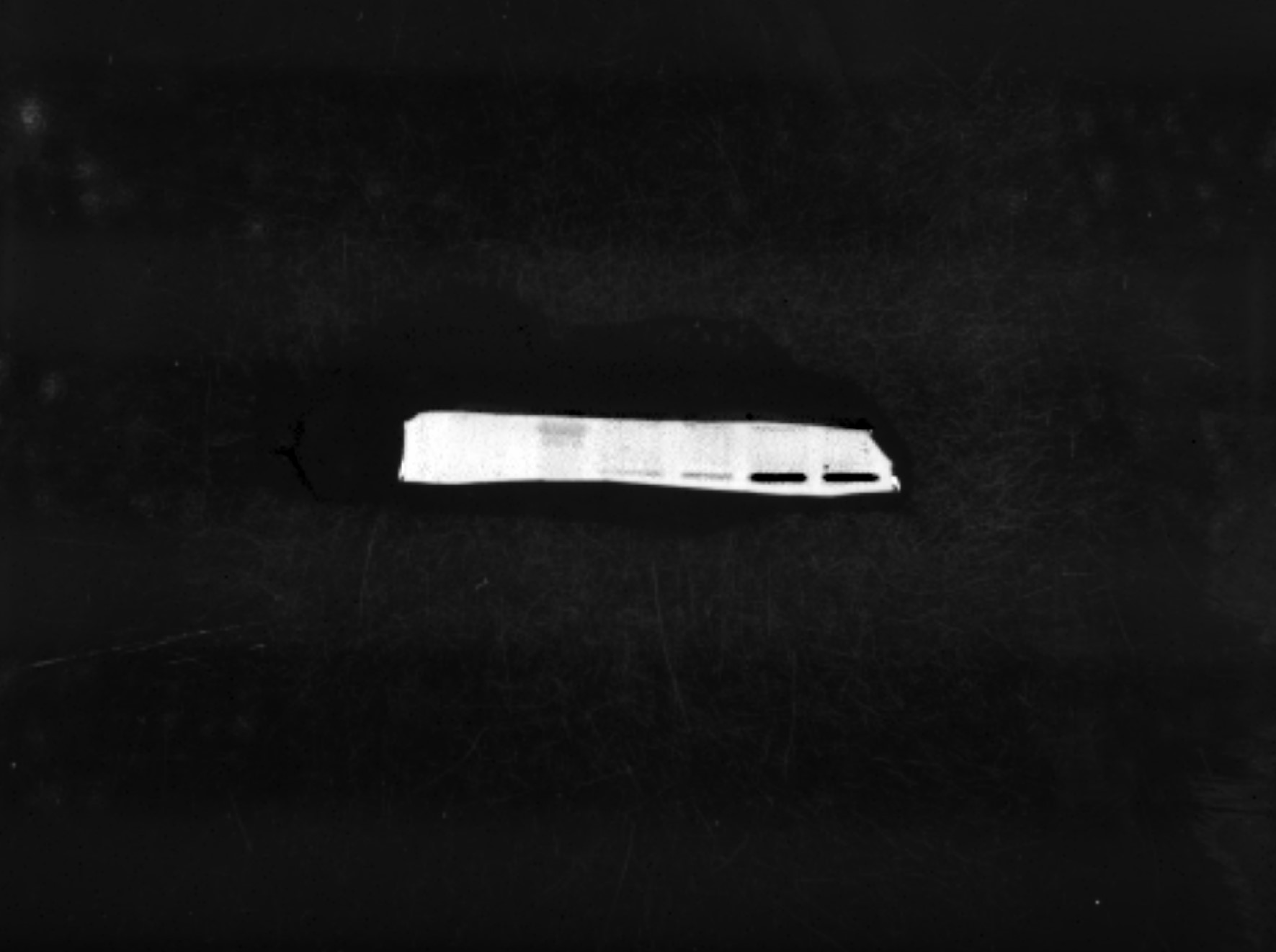

Supplement: Supplemental Information 4 [file peerj-09-10692-s004.zip › BCL-2-2.jpg]

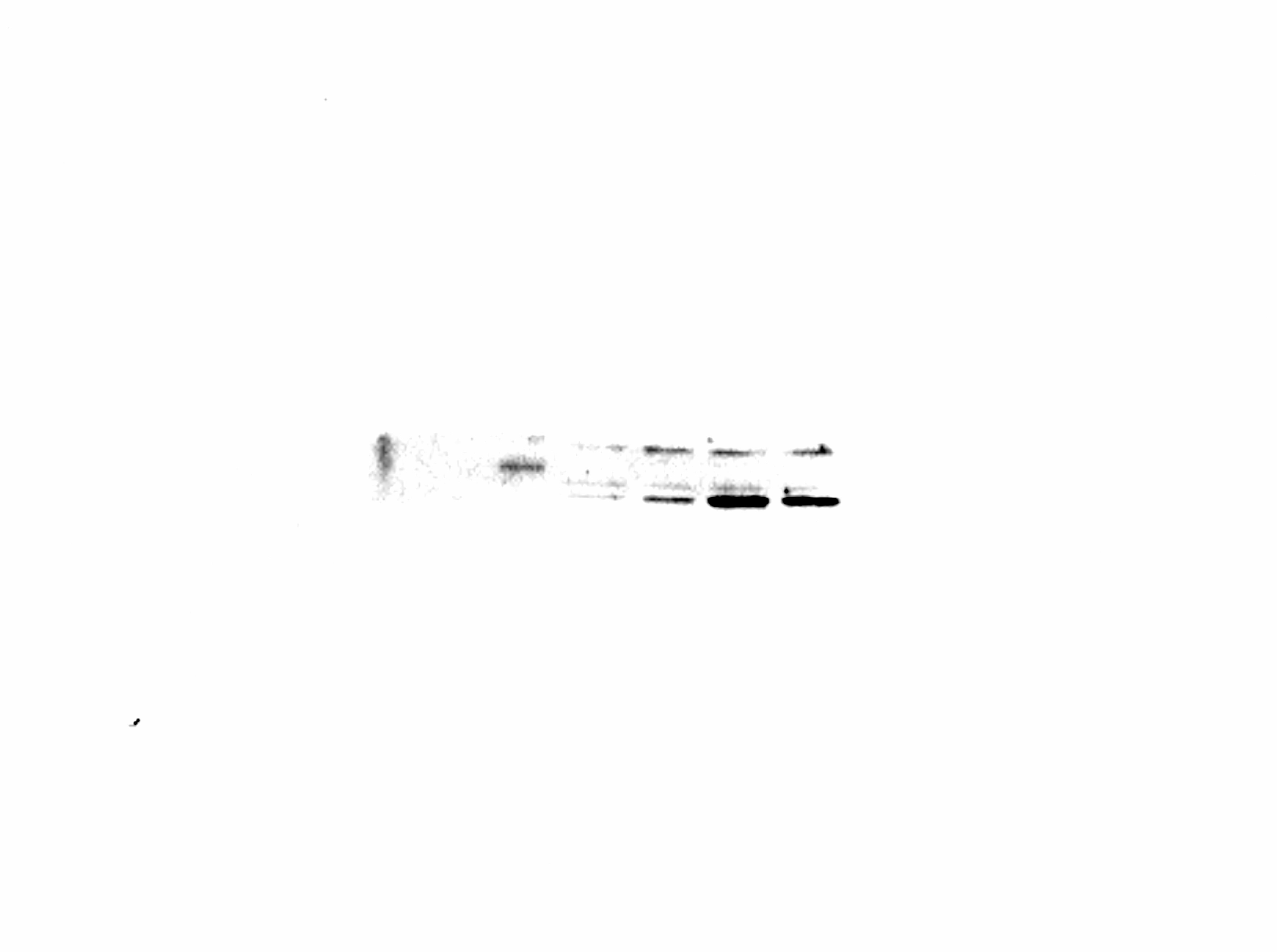

Supplement: Supplemental Information 4 [file peerj-09-10692-s004.zip › BCL-2-3.jpg]

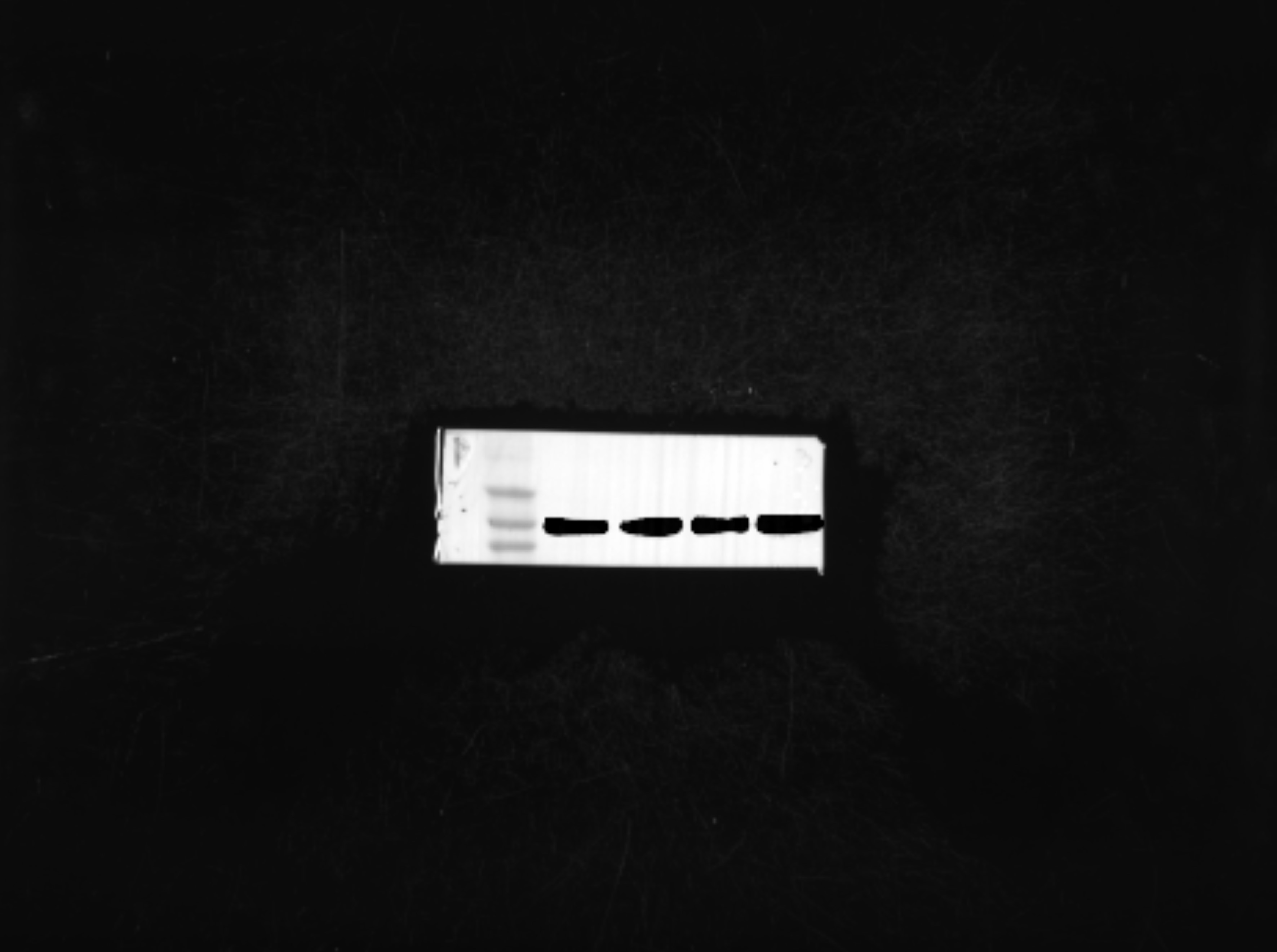

Supplement: Supplemental Information 4 [file peerj-09-10692-s004.zip › BAX-1.jpg]

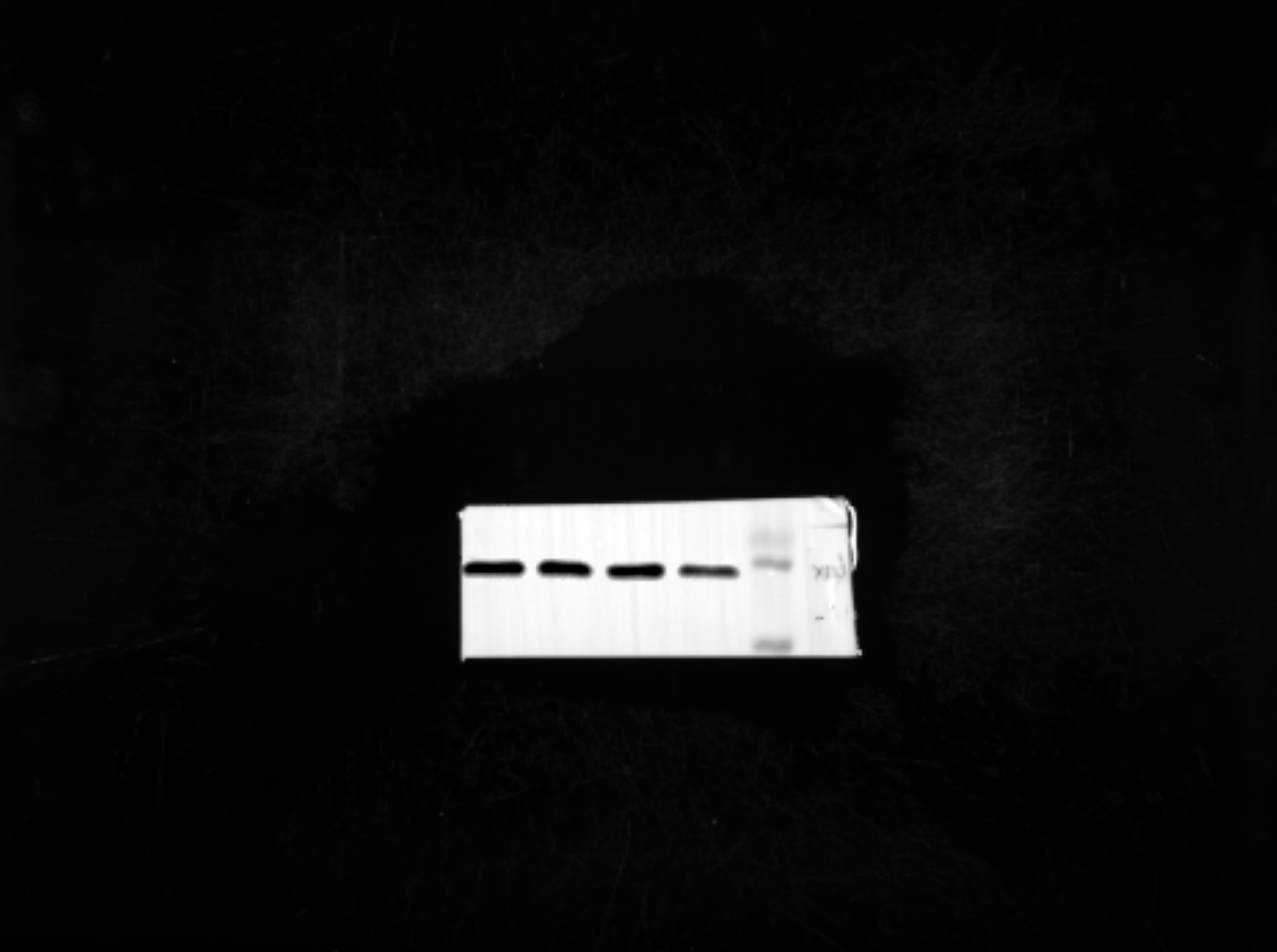

Supplement: Supplemental Information 4 [file peerj-09-10692-s004.zip › BAX-2.jpg]

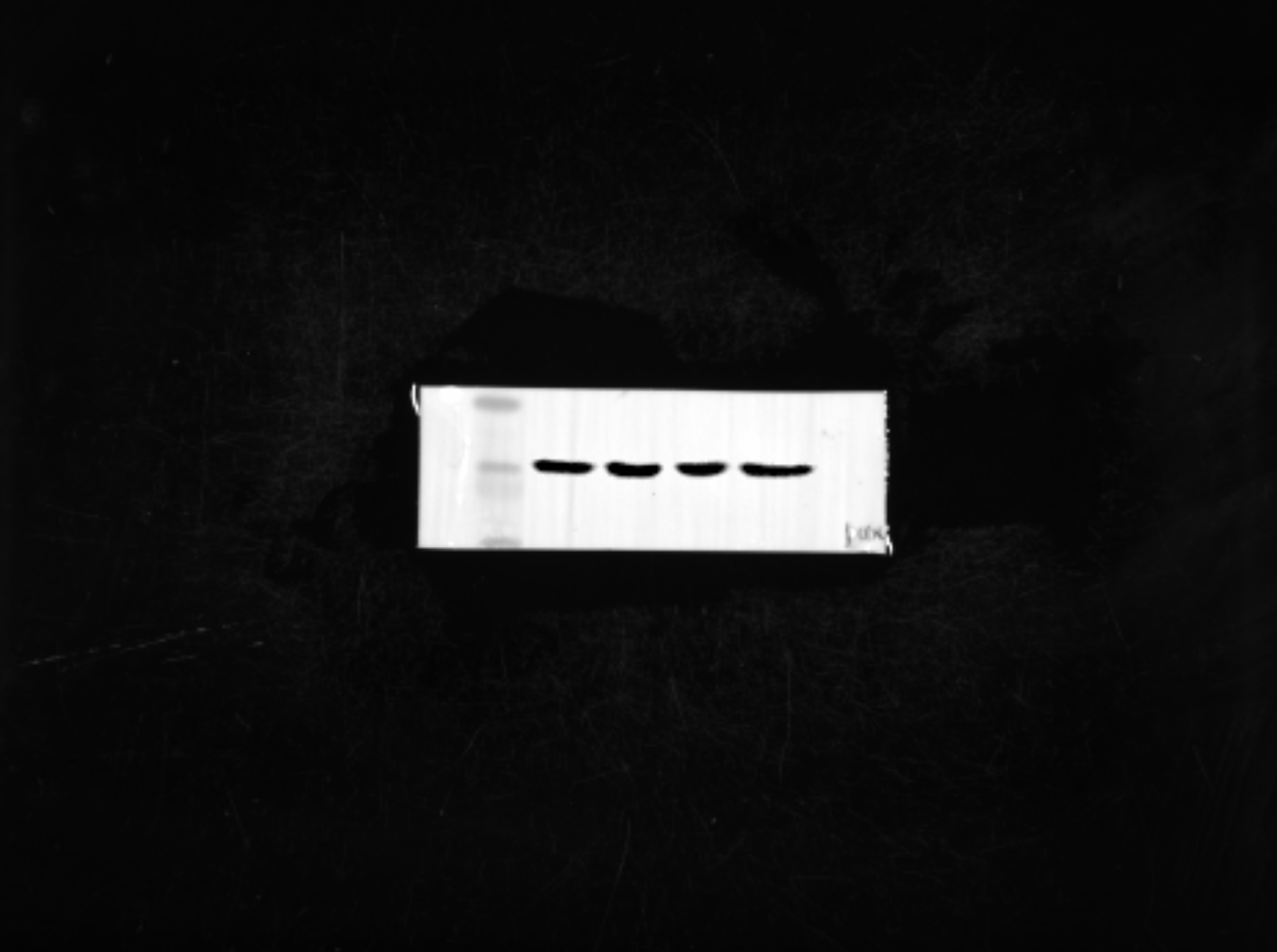

Supplement: Supplemental Information 4 [file peerj-09-10692-s004.zip › BAX-3.jpg]

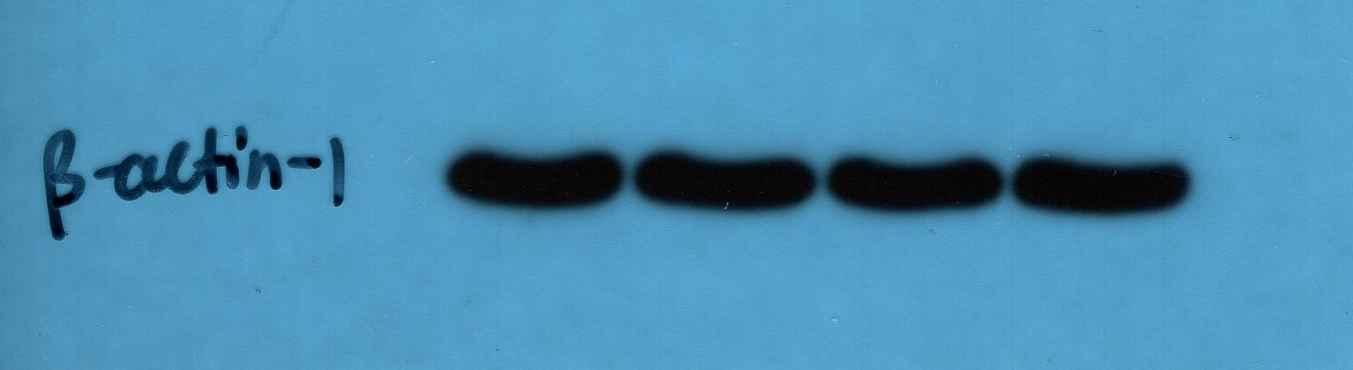

Supplement: Supplemental Information 4 [file peerj-09-10692-s004.zip › b-actin-1.jpg]

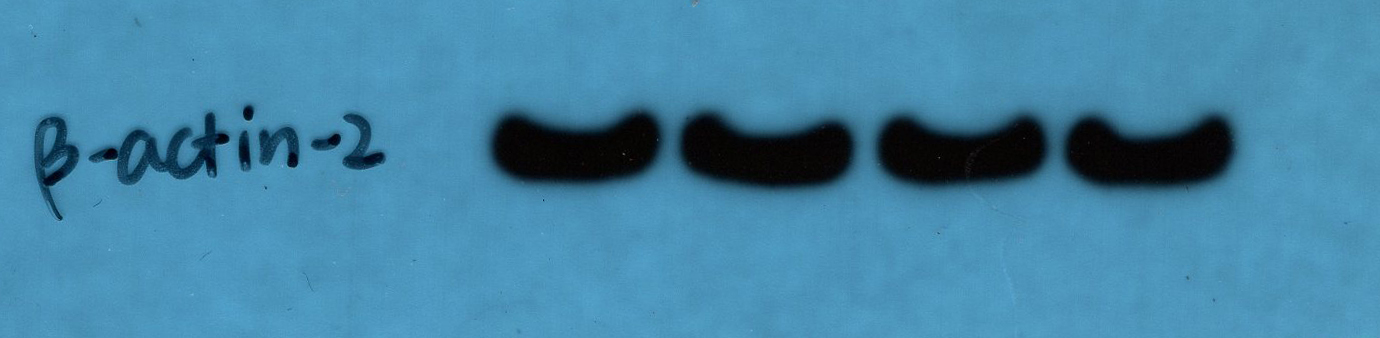

Supplement: Supplemental Information 4 [file peerj-09-10692-s004.zip › b-actin-2.jpg]

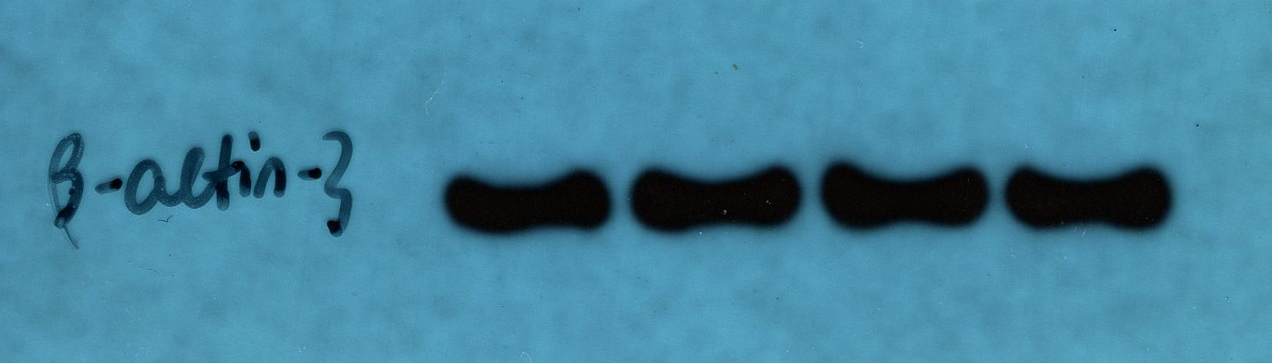

Supplement: Supplemental Information 4 [file peerj-09-10692-s004.zip › b-actin-3.jpg]

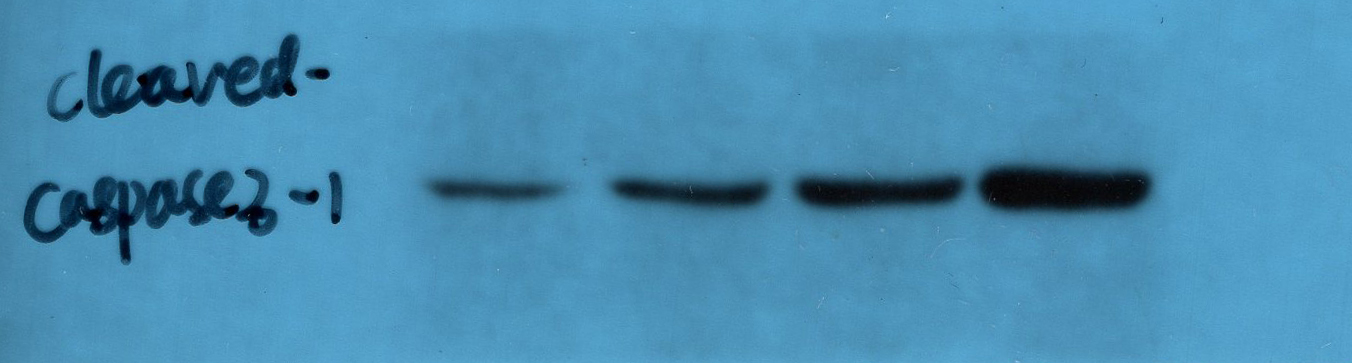

Supplement: Supplemental Information 4 [file peerj-09-10692-s004.zip › cleaved-caspase3-1.jpg]

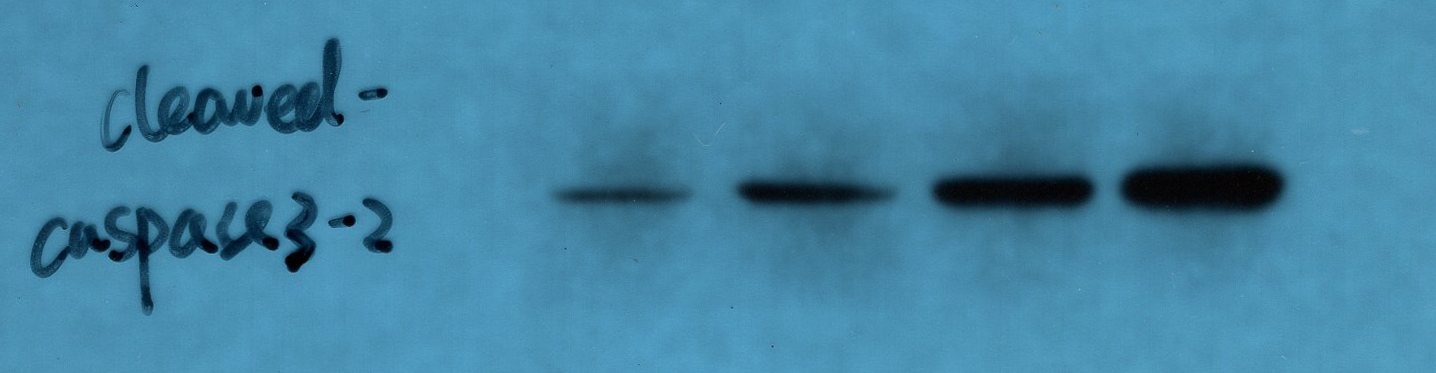

Supplement: Supplemental Information 4 [file peerj-09-10692-s004.zip › cleaved-caspase3-2.jpg]

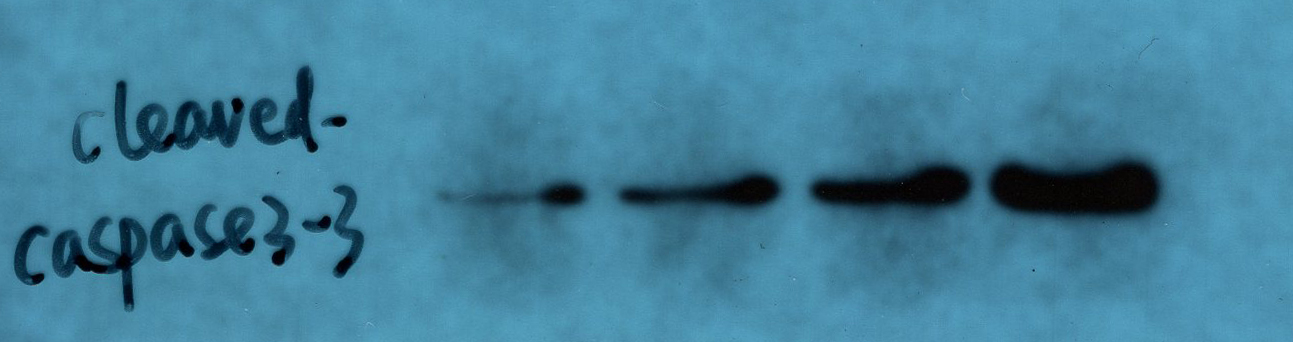

Supplement: Supplemental Information 4 [file peerj-09-10692-s004.zip › cleaved-caspase3-3.jpg]

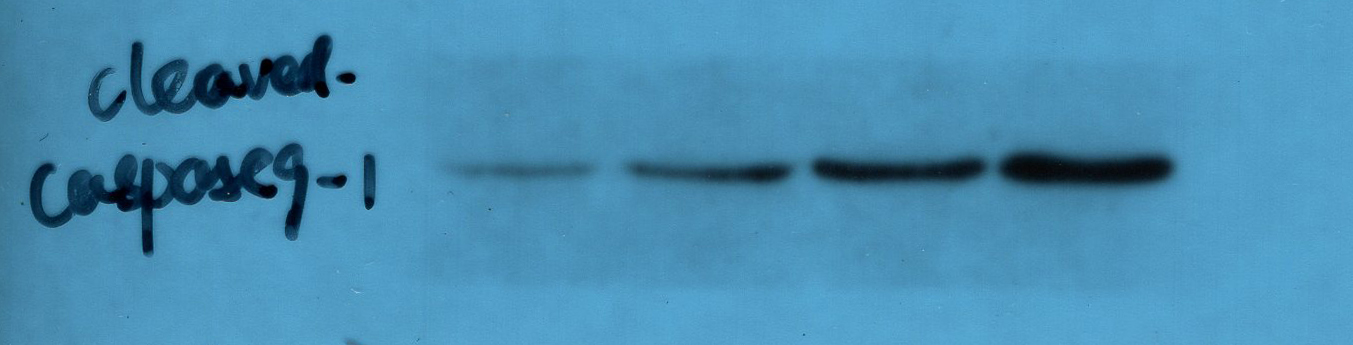

Supplement: Supplemental Information 4 [file peerj-09-10692-s004.zip › cleaved-caspase9-1.jpg]

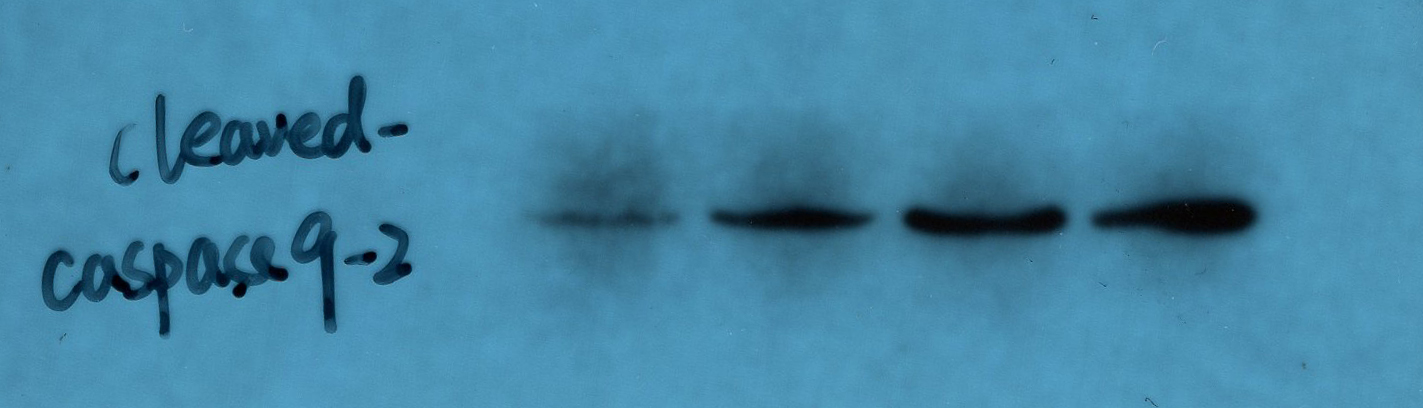

Supplement: Supplemental Information 4 [file peerj-09-10692-s004.zip › cleaved-caspase9-2.jpg]

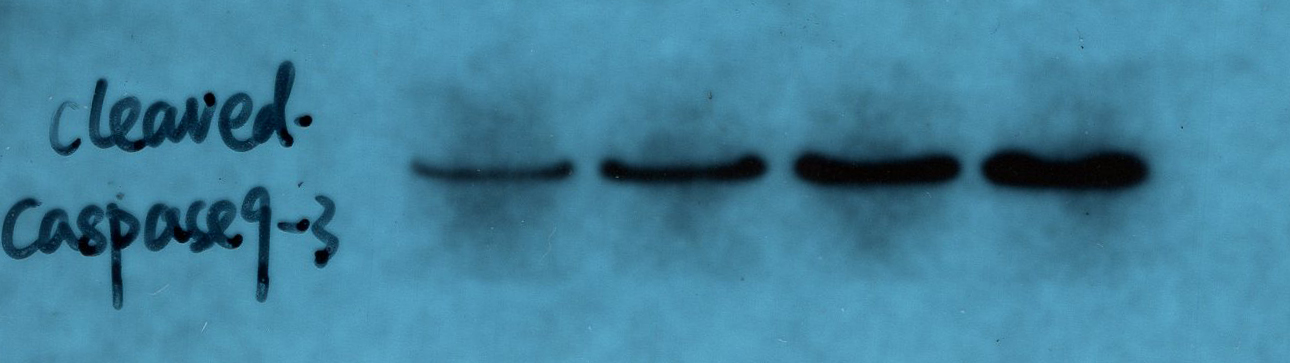

Supplement: Supplemental Information 4 [file peerj-09-10692-s004.zip › cleaved-caspase9-3.jpg]

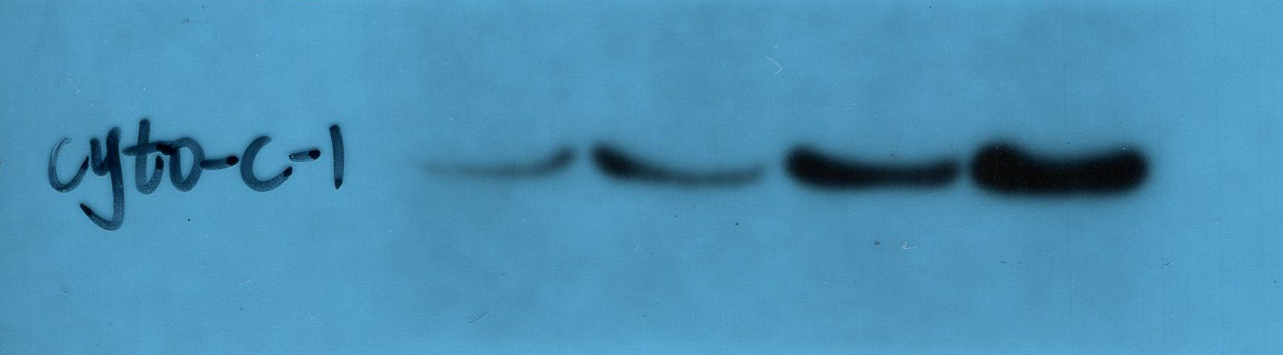

Supplement: Supplemental Information 4 [file peerj-09-10692-s004.zip › cyto-C-1.jpg]

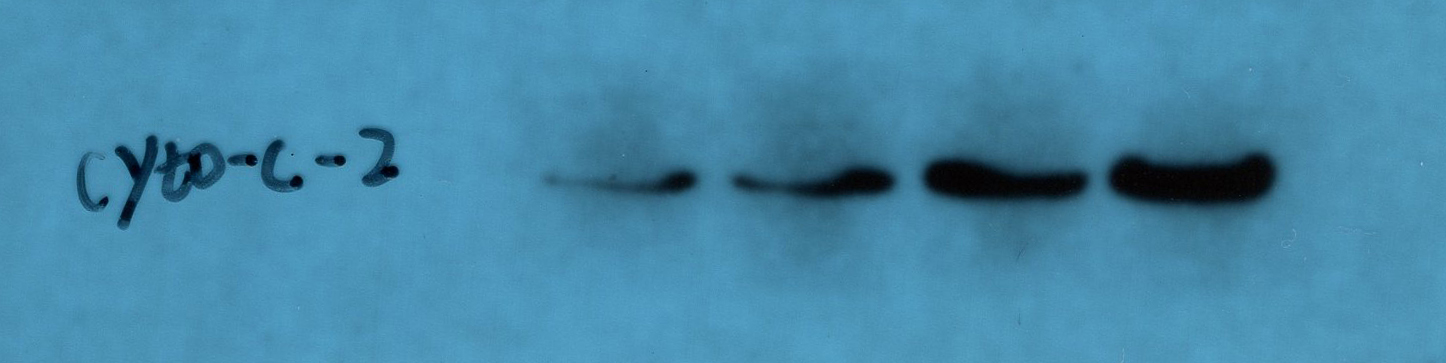

Supplement: Supplemental Information 4 [file peerj-09-10692-s004.zip › cyto-C-2.jpg]

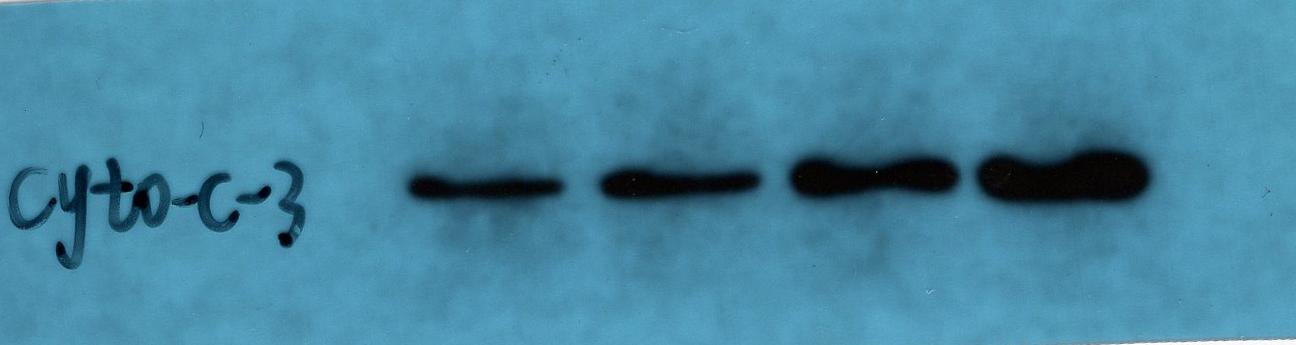

Supplement: Supplemental Information 4 [file peerj-09-10692-s004.zip › cyto-C-3.jpg]
