# Supplementary material for: Inhibitory effect and mechanism of action (MOA) of hirsutine on the proliferation of T-cell leukemia Jurkat clone E6-1 cells
Source: PeerJ. 2021 Feb 2;9:e10692. doi: 10.7717/peerj.10692 (PMC7863788; doi:10.7717/peerj.10692)

## Effects of hirsutine on Bax, Bcl-2, caspase 3 and caspase 9 gene expression in Jurkat Clone E6-1 cells

|  | DMSO | 50μM | 25μM | 10μM |
| --- | --- | --- | --- | --- |
| GAPDH | 19.50 | 24.46 | 23.46 | 20.42 |
|  | 19.13 | 24.11 | 23.89 | 20.59 |
|  | 19.36 | 24.14 | 23.44 | 20.42 |
|  |  |  |  |  |
| BCL-2 | 25.21 | 31.98 | 30.02 | 26.45 |
|  | 24.68 | 31.93 | 30.83 | 26.31 |
|  | 25.39 | 31.66 | 29.77 | 26.10 |
|  |  |  |  |  |
| Δt | 5.71 | 7.52 | 6.56 | 6.03 |
|  | 5.56 | 7.82 | 6.94 | 5.71 |
|  | 6.03 | 7.52 | 6.34 | 5.68 |
| 5.76 |  |  |  |  |
| ΔΔt | -0.06 | 1.75 | 0.79 | 0.26 |
|  | -0.21 | 2.06 | 1.18 | -0.05 |
|  | 0.27 | 1.75 | 0.57 | -0.09 |
|  |  |  |  |  |
| 2^-ΔΔt^ | 1.04 | 0.30 | 0.58 | 0.83 |
|  | 1.16 | 0.24 | 0.44 | 1.04 |
|  | 0.83 | 0.30 | 0.67 | 1.06 |
|  |  |  |  |  |
| mean | 1.01 | 0.28 | 0.56 | 0.98 |
| SD | 0.17 | 0.03 | 0.12 | 0.13 |

|  | DMSO | 50μM | 25μM | 10μM |
| --- | --- | --- | --- | --- |
| GAPDH | 19.50 | 24.46 | 23.46 | 20.42 |
|  | 19.13 | 24.11 | 23.89 | 20.59 |
|  | 19.36 | 24.14 | 23.44 | 20.42 |
|  |  |  |  |  |
| BAX | 24.54 | 29.54 | 28.10 | 25.31 |
|  | 24.69 | 28.11 | 28.89 | 25.29 |
|  | 24.29 | 28.75 | 28.02 | 25.56 |
|  |  |  |  |  |
| Δt | 5.04 | 5.08 | 4.64 | 4.90 |
|  | 5.56 | 4.01 | 5.00 | 4.70 |
|  | 4.93 | 4.61 | 4.59 | 5.14 |
| 5.17 |  |  |  |  |
| ΔΔt | -0.14 | -0.10 | -0.54 | -0.28 |
|  | 0.39 | -1.17 | -0.18 | -0.48 |
|  | -0.25 | -0.56 | -0.59 | -0.04 |
|  |  |  |  |  |
| 2^-ΔΔt^ | 1.10 | 1.07 | 1.45 | 1.21 |
|  | 0.77 | 2.24 | 1.13 | 1.39 |
|  | 1.19 | 1.48 | 1.50 | 1.03 |
|  |  |  |  |  |
| mean | 1.02 | 1.60 | 1.36 | 1.21 |
| SD | 0.22 | 0.60 | 0.20 | 0.18 |

|  | DMSO | 50μM | 25μM | 10μM |
| --- | --- | --- | --- | --- |
| GAPDH | 19.50 | 24.46 | 23.46 | 20.42 |
|  | 19.13 | 24.11 | 23.89 | 20.59 |
|  | 19.36 | 24.14 | 23.44 | 20.42 |
|  |  |  |  |  |
| CASP-3 | 29.53 | 33.57 | 33.92 | 31.92 |
|  | 30.99 | 33.21 | 33.78 | 31.69 |
|  | 30.60 | 33.20 | 33.62 | 31.68 |
|  |  |  |  |  |
| Δt | 10.03 | 9.12 | 10.46 | 11.51 |
|  | 11.86 | 9.11 | 9.90 | 11.10 |
|  | 11.24 | 9.06 | 10.18 | 11.25 |
| 11.04 |  |  |  |  |
| ΔΔt | -1.01 | -1.93 | -0.58 | 0.46 |
|  | 0.82 | -1.93 | -1.14 | 0.06 |
|  | 0.20 | -1.98 | -0.86 | 0.21 |
|  |  |  |  |  |
| 2^-ΔΔt^ | 2.02 | 3.80 | 1.50 | 0.72 |
|  | 0.57 | 3.82 | 2.21 | 0.96 |
|  | 0.87 | 3.95 | 1.81 | 0.86 |
|  |  |  |  |  |
| mean | 1.15 | 3.86 | 1.84 | 0.85 |
| SD | 0.76 | 0.08 | 0.36 | 0.12 |

|  | DMSO | 50μM | 25μM | 10μM |
| --- | --- | --- | --- | --- |
| GAPDH | 19.50 | 24.46 | 23.46 | 20.42 |
|  | 19.13 | 24.11 | 23.89 | 20.59 |
|  | 19.36 | 24.14 | 23.44 | 20.42 |
|  |  |  |  |  |
| CASP-9 | 29.26 | 32.31 | 32.38 | 30.08 |
|  | 28.81 | 32.09 | 32.04 | 30.54 |
|  | 29.29 | 32.04 | 32.06 | 29.87 |
|  |  |  |  |  |
| Δt | 9.76 | 7.85 | 8.92 | 9.67 |
|  | 9.68 | 7.99 | 8.15 | 9.95 |
|  | 9.93 | 7.90 | 8.62 | 9.45 |
| 9.79 |  |  |  |  |
| ΔΔt | -0.03 | -1.94 | -0.87 | -0.12 |
|  | -0.11 | -1.80 | -1.64 | 0.16 |
|  | 0.14 | -1.89 | -1.17 | -0.34 |
|  |  |  |  |  |
| 2^-ΔΔt^ | 1.02 | 3.84 | 1.82 | 1.09 |
|  | 1.08 | 3.49 | 3.11 | 0.90 |
|  | 0.91 | 3.71 | 2.24 | 1.27 |
|  |  |  |  |  |
| mean | 1.00 | 3.68 | 2.39 | 1.08 |
| SD | 0.09 | 0.18 | 0.66 | 0.19 |

## Primer information(Figure 6)

| **Gene name** | **Acession number** | **Fragment size（bp）** | **Primer name** | **Primer sequence (5'to3')** |
| --- | --- | --- | --- | --- |
| **BCL2** | **NM_000633.2** | **214** | **Forward** | **GACAACATCGCCCTGTGGAT** |
|  |  |  | **Reverse** | **GACTTCACTTGTGGCCCAGAT** |
| **Bax** | **NM_004324.4** | **223** | **Forward** | **GGCCCTTTTGCTTCAGGGTT** |
|  |  |  | **Reverse** | **AGCTGCCACTCGGAAAAAGA** |
| **caspase 3(CASP3)** | **NM_004346.4** | **182** | **Forward** | **TGGAACCAAAGATCATACATGGAA** |
|  |  |  | **Reverse** | **TTCCCTGAGGTTTGCTGCAT** |
| **caspase 9(CASP9)** | **NM_001229.5** | **193** | **Forward** | **AGGCCCCATATGATCGAGGA** |
|  |  |  | **Reverse** | **TCGACAACTTTGCTGCTTGC** |
| **GAPDH** | **NM_001289746.1** | **115** | **Forward** | **TCAAGAAGGTGGTGAAGCAGG** |
|  |  |  | **Reverse** | **TCAAAGGTGGAGGAGTGGGT** |

## PCR amplification curve(Figure 6)


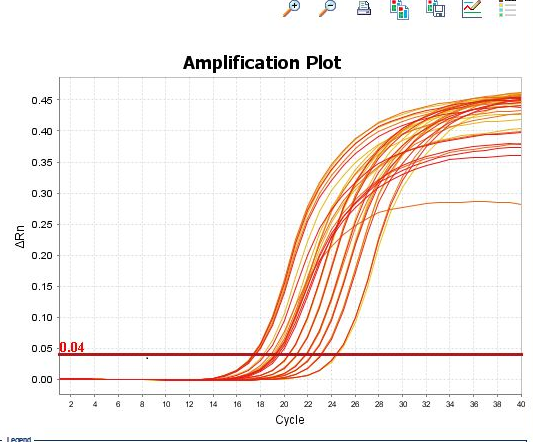

Supplement: Supplemental Information 5 [file peerj-09-10692-s005.docx]
